# Evaluation of Different Levels of Process Energy in Sorghum and Wheat-Based Diets on the Growth Performance of Pacific White Shrimp, *Litopenaeus vannamei*

**DOI:** 10.1155/anu/1985325

**Published:** 2024-11-25

**Authors:** Tucker Graff, Trinh Ngo, Donald Allen Davis, Sajid Alavi

**Affiliations:** ^1^Department of Grain Science and Industry, Kansas State University, Manhattan, Kansas 66506, USA; ^2^School of Fisheries, Aquaculture and Aquatic Sciences, Auburn University, Auburn, Alabama 36849, USA

**Keywords:** digestibility, gelatinization, Pacific white shrimp, starch, thermal energy

## Abstract

This research evaluated the effect of different levels of extrusion process energy during the production of wheat and sorghum-based feeds on the growth performance and digestibility of Pacific white shrimp (*Litopenaeus vannamei*). Process energy consisted of mechanical and thermal components, which were both modulated via varying preconditioner steam addition. Diets were formulated to be isonitrogenous (36% protein) and isolipidic (8% fat), with three levels of thermal energy (TE) input during preconditioning (high, medium, and low). All diets showed increased starch gelatinization with increased TE, with the wheat-based diets undergoing a greater degree of gelatinization than the sorghum-based diets. There were no significant differences in final biomass, weight gain, feed conversion ratio (FCR), or survival among the different treatments. However, digestibility results showed that wheat-based diets had significantly higher apparent digestibility coefficients (ADCs) for both protein and energy compared to sorghum-based diets. Overall, increased starch gelatinization that correlated with greater digestibility was observed, but this effect was reduced in the sorghum-based diets. These findings suggest that sorghum can be used as a viable alternative to wheat in shrimp feed without negatively impacting their growth performance, while also offering potential cost savings to producers.

## 1. Introduction

Pacific white shrimp (*Litopenaeus vannamei*) is one of the top-produced aquaculture species requiring a high-quality, water-stable pellet [1]. Historically, feed has accounted for 50%–70% of the total cost of production [2]. Carbohydrates (CHO), such as the starches found in cereal grains, are typically included in formulations even though aquatic animals do not have any specific requirements for carbohydrates [3]. Increasing dietary carbohydrates has been used as a strategy to minimize the use of dietary protein, as carbohydrates can act as an immediate energy source for many crustaceans [4]. Research has shown that carbohydrates have a dietary protein-sparing effect [5, 6]. This protein-sparing effect of increasing dietary carbohydrates can help significantly reduce feed costs as carbohydrates are the least expensive nutrient for aquatic animals that are energy yielding [2]. Carbohydrate sources can also act as a binder that improves various pellet qualities; most notably water stability and pellet durability [7].

Feed manufacturers typically select carbohydrate-rich ingredients based primarily on cost and availability, often without considering the potential effects on shrimp growth. Cruz-Suárez et al. [5] tested diets with wheat meal, corn flour, and sorghum flour, and they found that the wheat-based diet resulted in the best growth rate, with corn and sorghum flour being the least efficient. Those authors noted, however, that cheaper carbohydrate sources such as sorghum can overcome the economic impact of slightly higher FCRs in terms of shrimp production cost. Similarly, a literature review by Cuzon et al. [6] found that wheat starch was highly digestible (90%) by *L. vannamei*, further proving its suitability as a source of energy.

Another consideration for the choice of CHO source is its ability to act as a pellet binder; this comes from the native starch present in the grain, and in the case of wheat, the presence of gluten. Feeds formulated with wheat starch showed improved pellet-forming ability, water stability, and lesser amounts of fines during handling [8]. Similarly, vital wheat gluten inclusion was shown to yield a strong pellet with a regular cell structure that yielded a smooth, nonporous surface [9]. The presence of gluten in wheat has led to wheat starch being the preferred CHO in shrimp feed as it improves pellet quality. However, wheat is often more expensive than other cereal grains and requires higher inputs such as irrigation and nitrogen application, increasing its environmental impact.

As water becomes scarcer in high-production regions, it will become necessary for feed producers to reduce their reliance on costly cereal grains such as wheat. Grain sorghum is a non-genetically modified organism (GMO) cereal crop with improved drought tolerance when compared to wheat, which suffers extreme yield loss and crop failure when undergoing periods of drought and water stress [10]. At low water use, sorghum has been shown to have better yields per acre than maize [11]. It is one of the top 5 cereal crops globally, with the largest producer being the United States (62.3 million tons) in 2020 [12]. Grain sorghum has a high starch content (70%–72%), protein (11%–12.8%), and total dietary fiber (8.4%–10.9%) [13]. This high starch content makes sorghum an attractive choice for extruded feed applications as starch serves as the primary binder in extruded feeds, helping to bind the pellet together, and prevent disintegration as it is submerged in water [3].

Extrusion processing has quickly become the primary method for producing feed for aquaculture. Compared to older technologies such as pellet milling, extruded feed experiences much higher levels of specific thermal energy (STE), pressure, and moisture levels [14]. This change in processing conditions leads to increased levels of gelatinization in starch, increasing the availability of carbohydrates when consumed. Energy is transferred to raw material through both specific mechanical energy (SME) and STE. Extrusion processing includes preconditioning, extrusion using mechanical screws, and cutting with a rotational knife [15]. Preconditioning helps ensure diet uniformity through continuous mixing of the raw material with steam and water. This preheats the material and begins the cooking process and starch gelatinization, which is then finalized using mechanical screws to generate SME [16]. STE occurs primarily in the preconditioner through liquid water and steam addition [17, 18]. This helps ensure improved final product quality; by starting the process of starch gelatinization in the preconditioner, raw material granules are hydrated and softened. This plasticization of the raw diet helps increase extruder stability, which in turn leads to improvements in final product quality [19, 20].

An English-based literature review identified a few publications connecting processing and shrimp performance and one pertaining to the impact of varying steam addition levels on the growth rates and digestibility of Pacific white shrimp [21]. Given that carbohydrates can act as an energy source for crustaceans and that gelatinization increases the availability of carbohydrates when consumed, the hypothesis of this study was that increasing the total amount of process energy via preconditioner steam addition would result in increased digestibility of the feed when consumed. Process energy consists of STE and SME, which were both modulated by varying the amounts of preconditioner steam addition. Therefore, the objective of this study was to evaluate the impact of three levels of process energy in both wheat and sorghum-based diets on the digestibility and growth rates of Pacific white shrimp.

## 2. Materials and Methods

### 2.1. Experimental Design and Diets

A 6-week growth trial was conducted to compare the effects of different process energy inputs (high, medium, and low) during extrusion of sorghum and whole wheat-based practical diets for white shrimp. This constituted a 3 × 2 factorial design with a total of six experimental treatments. Total process energy inputs were modulated by varying the preconditioner steam input which impacted both SME and STE levels. Diets were formulated to be isonitrogenous (36% protein) and isolipidic (8% lipid). Diet formulations are presented in Table 1. Titanium dioxide was included in the dry mix at 0.6% inclusion for the digestibility trial before extrusion of the feeds. The experimental diets were prepared with a pilot-scale single screw extruder (Model ×20, Wenger Manufacturing, Inc., Sabetha, KS, USA) at Kansas State University (Manhattan, KS, USA) using standard procedures for shrimp feeds. Diets were top coated with 2% fish oil and 0.12% cholesterol prior to feeding. Proximate composition analysis and amino acid (AA) composition were analyzed for all experimental diets at MidWest Laboratories (Omaha, NE, USA) and University of Missouri Agricultural Experiment Station Chemical Laboratories (Columbia, MO, USA) (Tables 2 and 3, respectively). Starch gelatinization was analyzed for all diets in triplicate at MidWest Laboratories (Omaha, NE, USA) and is shown in Table 4.

### 2.2. Growth Trial

A growth trial took place at E.W. Shell Fisheries Center (Auburn University, Auburn, AL, USA). During the growth trial, six replicate groups for each of the six dietary treatments and four replicate groups for the commercial diet were then randomly assigned and shrimp were fed four times per day. Fifteen postlarvae shrimp were hand-sorted to uniform size and stocked into each aquarium tank (120 L, 52 × 52 × 49.5 cm) in the experimental system. A total of 40 aquarium tanks were used in the study. The total amount of shrimp per tank was counted weekly to adjust the survival rate. The daily feeding ration was calculated using our historical data of estimated weekly weight gain, expected feed conversion ratio (FCR) of 1.6, the survival rate of the corresponding week, and the actual feeding response of the animal [23]. At the end of the growth trial, shrimp were counted, and group weighed to determine mean final biomass, final weight, survival, and FCR according to standard calculations [24]. Upon termination of the trial, shrimp were weighed and counted and five individuals per tank were randomly selected, packed in sealed bags, and stored in a freezer (−20°C) for chemical analysis. Shrimp samples were dried in an oven at 90°C to a constant weight, using the methods described by the Association of Official Agricultural Chemists (AOAC) [22], and then ground in a coffee grinder and stored. Total energy content was determined using a microcalorimetric adiabatic bomb using benzoic acid as standard (Parr 6725, Moline, IL, USA) [25].

### 2.3. Digestibility Trial

The digestibility trial was performed in a recirculating aquaculture system comprised of a battery of 80 L glass tanks connected to a common reservoir, a biological, and a physical filter. Additionally, a charcoal cartridge filter with 10-inch 50 μm wound polypropylene filter cartridge (Pentair plc [PNR], Golden Valley, MN, USA) was supplemented to the system to adsorb any particulates that might contribute to water quality issues. During the entire trial, water quality parameters were maintained within acceptable ranges for shrimp culture [26].

Nine shrimp (average initial individual weight of 30.33 ± 1.98 g) were stocked into 36 glass aquaria of the abovementioned system. Six replicate aquaria were offered one of the six experimental diets, and the resulting fecal material from each two tanks was pooled in a total of three replicate fecal samples/diet. The shrimp were acclimated to experimental diets for 3 days prior to fecal material collections. Feces were collected four times a day for a period of three consecutive days. Shrimp were offered feed in the morning, and then after 1 h, the first collection of feces was discarded. The subsequent fecal collections were rinsed with distilled water and oven-dried for 24 h prior to storage until analyzed. Gross energy of the diets and the fecal material was analyzed using a semimicro calorimeter (Model 6725, Parr Instrument Co., Moline, IL, USA). Titanium dioxide concentration was determined according to Short et al. [27], in which samples were ashed, then digested using 7.4M sulfuric acid, then adding hydrogen peroxide, resulting in yellow-orange coloration. After 48 h of incubation, the absorbance of the samples was read using a spectrophotometer at 410 nm (Genesys 20, Thermo Fisher Scientific, Waltham, MA, USA), and titanium dioxide concentration was determined using the equation from the standard curve. Samples were sent for protein and AA analysis in the laboratories of the University of Missouri (University of Missouri Agricultural Experiment Station Chemical Laboratories, Columbia, MO, USA). The apparent digestibility coefficients (ADCs) for protein (APD), and energy (AED) of the diets (D) were calculated according to Cho, Slinger, and Bayley [28] as follows:

$ADP\;and\;AED\;\left(\%\right)=100-\left[100\times \left(\frac{TiO_{2}\;in\;feeds\;\left(\%\right)}{TiO_{2}\;in\text{ feces }\left(\%\right)}\times \frac{\text{nutrients }in\text{ feces }\left(\%\right)}{\text{nutrient }in\text{ feeds }\left(\%\right)}\right)\right].$

### 2.4. Water Quality

Dissolved oxygen (DO) was maintained near saturation using air stones in each culture tank via a common airline connected to a regenerative blower (Model R4P115, Gast Manufacturing, Benton Harbor, MI). DO, salinity, and water temperature were measured twice daily using a YSI-2030 Pro digital oxygen/temperature meter (YSI Corporation, Yellow Springs, Ohio, USA), and total ammonia N (TAN) and nitrite-N were measured twice per week using YSI 9300 photometer (YSI, Yellow Springs, OH, USA). The acidity (pH) of the water was measured two times per week during the experimental period using a pHTestr30 (Oakton Instrument, Vernon Hills, IL, USA) (Table 5).

### 2.5. Statistical Analysis

All data were analyzed using Statistical Analysis System (SAS) (V9.4, SAS Institute, Cary, NC, USA). Growth indices and ADCs of shrimp were analyzed using two-way analysis of variance (ANOVA) to determine significant differences (*p*  < 0.05) among treatments followed by Tukey's multiple comparison test to evaluate significant differences between treatment means.

## 3. Results and Discussion

### 3.1. Proximate Analysis

The proximate composition and AA profile of the extruded diets are shown in Tables 2 and 3. Protein and fat contents of the diets ranged from 37.70% to 38.50% and 6.90% to 7.22%, respectively, with formulation called for 36% protein and 8% fat. There was a slight increase in AA content in the low STE diets, but their values were minimal.

### 3.2. Starch Gelatinization

Gelatinization data for the extruded diets ranged from 83.00% to 98.75% and are shown in Table 4. In both grains, gelatinization increased as more process energy was used in the manufacturing of the diets. These differences were significant; sorghum-based diets were significantly different from all wheat-based diets with the exception of the high-energy sorghum diet (90.49%). All wheat-based diets ranged from 94.56% to 98.75%; there were no significant differences between the high-energy diet and the low-energy diet, indicating that the impact of process energy inputs is less for wheat than it is for sorghum. Part of the change in process energy between treatments was due to an increase in STE contributed by steam during preconditioning. Research on STE in extrusion is limited, but these results were also seen by Pacheco et al. [18] in dog food; results there showed that as STE during extrusion increased via steam addition, so did the gelatinization of the kibble. Dietary composition can affect the degree of starch gelatinization due to differences in starch source and also interactions with other dietary components [29, 30]. This might have been a factor in the present study as can be seen from the lower degree of gelatinization in the sorghum-based diets as compared to the wheat-based diets. Temperature and water content are some of the processing factors affecting gelatinization in starch [31]. Although there was no difference between sorghum and wheat-based diets with regard to independently controlled extrusion conditions including moisture, higher process temperatures were observed for the former due to raw material steam absorption differences [32]. However, sorghum-based feeds still had lower overall starch gelatinization than wheat-based feeds. Sorghum starch is more resistant to gelatinization as compared to other grains due to the surrounding protein matrix in the endosperm that acts as a barrier, although the starch granules are similar to those in other cereals such as corn in terms of shape and size [33–35]. Wheat starch has a gelatinization temperature range of 58−64°C, while sorghum has a gelatinization temperature range of 68−76°C [34, 36] indicating that either more heat or longer exposure to increased temperatures is necessary to achieve full gelatinization in sorghum-based diets. It should be noted, however, that these gelatinization temperature ranges are based on a calorimetric technique, whereas the gelatinization data reported in this study were based on enzymatic digestion. Similar results were observed in a previous study that reported lower gelatinization (84%–86%) for sorghum-based dry extruded cat food as compared to rice and corn-based cat food (89%–90%), based on the enzymatic method [37].

### 3.3. Growth Trials

The growth trial was performed without any significant water quality or disease issues. Water quality was within suitable parameters for the culture of Pacific white shrimp, as shown in Table 5. No significant differences were observed in final biomass, final mean weight, weight gain, feed conversion ratio (FCR), survival, gross energy, and energy retention when Pacific white shrimps were fed with diets containing different wheat and sorghum process energy input via preconditioner steam addition (*p*  > 0.05), as shown in Table 6. There were no significant differences between diets based on grain source used and process energy, and there was no interaction between them. Limited studies with sorghum in Pacific white shrimp diets exist; limited studies with sorghum in Pacific white shrimp diets exist; inclusion of sorghum DDGs in both extruded and pelleted diets was studied with results showing successful incorporation into practical diets for Pacific white shrimp at levels up to 40% in a clear water system without negatively affecting growth, survival, and FCR [38, 39]. However, this is not a straight comparison, as sorghum DDGs were used as a protein source and a replacement for soybean meal rather than a primary carbohydrate source. Sorghum DDGs were subjected to processing during the ethanol manufacturing process, with much of the starch being removed, so their final composition had much higher protein, fiber, and lipids (34%, 10%, and 9%, respectively) and lower starch (24%) than grain sorghum. Other research has, however, shown that omnivorous fish such as tilapia can consume sorghum-based diets with no adverse effects on growth rates. Tilapia fed a diet of 25% grain sorghum or up to 30% sorghum starch had increased growth performance in comparison to tilapia fed corn, wheat, barley, and rice [40, 41].

### 3.4. Digestibility Trials

**Figure 1:**
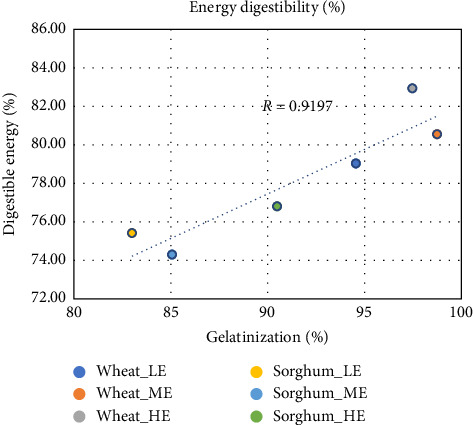
Linear regression of digestibility and starch gelatinization for all treatments.

**Figure 2:**
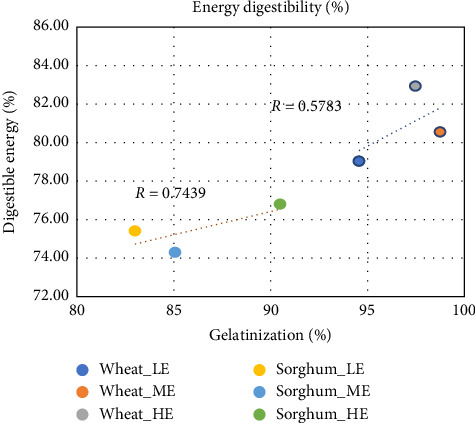
Linear regression of digestibility and starch gelatinization based on grain type.

Apparent protein and energy digestibility coefficients (APDD and AEDD, respectively) are shown in Table 7, and energy digestibility results are also plotted with respect to starch gelatinization in Figures 1 and 2. ADCs of energy ranged from 74.29% to 82.93%. Overall, there is a strong relationship between digestible energy and degree of gelatinization (*r* = 0.9197) indicating that a greater degree of starch gelatinization resulted in a greater amount of digestible energy, as shown in Figure 1. However, when the data were broken down according to grain type, differences were observed between wheat and sorghum-based feeds; starch gelatinization and digestible energy had a stronger correlation in sorghum-based feeds (*r* = 0.7439) than in wheat-based feeds (*r* = 0.5783), as shown in Figure 2. Starch digestibility in sorghum is complex; it can be affected by the starch–protein interaction, antinutritional factors such as tannins (although the sorghum used in the current study was tannin-free), and nonstarch polysaccharides such as cellulose or hemicellulose [33, 35]. Due to these reasons, sorghum starch is not as readily gelatinized as wheat as was stated previously. But increasing amounts of process energy resulted in a significant impact on degree of gelatinization in sorghum-based feeds (increase from 83% to 90.5%) and consequently on digestible energy as well. In the case of wheat-based feeds, gelatinization range was already fairly high (94.5%–98.7%) irrespective of the energy input, so the impact on digestible energy was less. Another potential explanation might be the high water stability of the wheat-based pellets irrespective of process energy input resulting in less loss of nutrients before the feed is consumed, while sorghum-based diets had a significant improvement in water stability with higher process energy [32]. The latter was related to the increase in gelatinization and consequently binding ability of starch. Thus, in the case of sorghum, two interlinked reasons led to a higher correlation between starch gelatinization and energy digestibility as compared to wheat; one, the degree of starch susceptibility due to gelatinization and, two, water stability.

Significant differences for both AEDD and APDD did exist between grain source (*p*  < 0.001), and between diets with different levels of process energy (*p*=0.001), but no interaction between grain source and process energy existed (*p*=0.056). Significantly higher levels of AEDD were reported in wheat-based diets (79.03%–82.93%) than in sorghum-based diets (74.29%–76.79%). Apparent digestible energy (ADE) values of 77.1% and 76.2% for wet-extruded wheat and sorghum-based diets, respectively, were reported by Davis and Arnold [42]. This is in line with the degree of gelatinization results in this research as well. Wheat-based diets were overall more gelatinized than the sorghum-based diets; this resulted in a higher amount of energy digested during consumption. AEDD values increased in both grain types as TE input increased. These trends held true for the apparent protein digestibility of the diets as well; wheat-based diets had an APDD of 86.99%–82.59%, and sorghum-based diets had an APDD of 79.81%–77.29%. In both diets, higher TE input levels result in an increased digestibility coefficient. The lower energy and protein digestibility of sorghum-based diets is likely due to the protein being tightly bound in the endosperm, and the starch being relatively inaccessible because of the surrounding protein matrix acting as a barrier, as was discussed earlier.

Limited research exists in aquaculture regarding the digestibility of sorghum-based diets. A 2006 study with carp reported a protein digestibility value of 71.86% in carp [43]. Conversely, a study with sunshine bass showed lower sorghum protein digestibility values of 60% [44]. It is likely that these differences are likely due to processing methods; sorghum starch is bound in a protein matrix which will limit the ability of enzymes to access and digest it. Processing methods, such as extrusion, help to release the starch from the protein matrix and increase the digestion of sorghum [45]. Studies have shown that extrusion enhances the protein digestibility of sorghum by 18% or a maximum of 30% [46, 47]. The amount of process energy appeared to also help with this based on data from the current study; as process energy input levels increased via increased steam addition, the degree of starch gelatinization also increased, indicating that more of the starch was released from the protein matrix, which in turn correlated to a higher AEDD and APDD in both diets.

## 4. Conclusion

The results of this study indicate that grain sorghum can be used in practical diets for Pacific white shrimp as a replacement for whole wheat without any negative effects on growth, survival, and FCR. Results also show that reduced process energy inputs via reduced preconditioner steam addition do not significantly impact growth rates, creating possible cost-saving opportunities for feed producers. Whole wheat-based diets showed higher apparent digestibility values for both protein and energy in comparison to sorghum, especially in high process energy diets. Based on these observed results, sorghum can be used as a carbohydrate source in shrimp cultivation.

## Figures and Tables

**Table 1:** Diet formulation and proximate analysis of shrimp diets containing different wheat and sorghum process energy input.

Ingredient (%)	Whole wheat	Sorghum
Fishmeal	10.00	10.00
Soybean meal	39.00	39.45
CPC^a^	8.00	8.00
Fish oil	2.00	2.00
Soy oil	3.02	2.65
Lecithin	1.00	1.00
Cholesterol^b^	0.12	0.12
Whole wheat	23.20	0.00
Rice Bran	10.00	10.00
Sorghum^c^	0.00	23.12
Mineral premix^d^	0.07	0.07
Vitamin premix^d^	0.04	0.04
Choline chloride	0.20	0.20
Stay-C 35% active	0.10	0.10
CaP-dibasic	1.75	1.75
Bentonite	1.50	1.50

*Note:* All other ingredients were sourced through Fairview Mills (Seneca, KS, USA).

^a^Cargill, Inc., Wayzata, MN, USA.

^b^MP Biomedicals Inc., Solon, OH, USA.

^c^Nu-Life Market, Scott City, KS, USA.

^d^Ziegler Bros, Inc., Gardners, PA, USA.

**Table 2:** Proximate composition of practical shrimp diets with different processing energy of sorghum and wheat.

Proximate composition (as is)	Wheat_LE	Wheat_ME	Wheat_HE	Sorghum _LE	Sorghum _ME	Sorghum_HE
Moisture (%)^a^	5.47	6.06	6.47	5.64	6.13	6.12
Crude protein (%)^b^	37.85	37.70	38.10	38.05	38.50	38.05
Crude fat (%)^c^	7.22	7.13	7.16	7.02	6.90	6.98
Fiber (%)^d^	3.20	2.90	2.85	3.20	2.75	3.05
Ash (%)^e^	10.35	10.15	10.20	10.40	9.92	9.88
Sulfur (%)	0.39	0.38	0.38	0.39	0.39	0.40
Phosphorus (%)	1.25	1.24	1.24	1.26	1.20	1.26
Potassium (%)	1.31	1.31	1.31	1.33	1.29	1.40
Magnesium (%)	0.29	0.29	0.28	0.30	0.28	0.31
Calcium (%)	1.27	1.28	1.27	1.28	1.23	1.27
Sodium (%)	0.10	0.09	0.09	0.09	0.09	0.10
Iron (ppm)	217.50	220.50	223.50	234.00	237.00	227.50
Manganese (ppm)	85.80	84.05	90.15	81.50	76.85	80.20
Copper (ppm)	38.90	39.55	44.05	41.00	39.15	41.35
Zinc (ppm)	79.80	78.50	86.95	77.10	75.75	79.95

*Note:* All minerals were tested using AOAC 981.01. Diets were made at Kansas State University. Values represent means of two replicates. Analysis was performed by Midwest Laboratories, Inc., Omaha, NE, USA.

Abbreviations: AOAC, Association of Official Agricultural Chemists; HE, high process energy; LE, low process energy; ME, medium process energy.

^a^AOAC 930.15.

^b^AOAC 990.03.

^c^AOAC 954.02 (via acid hydrolysis).

^d^AOCS Ba-6a-05.

^e^AOAC 942.05.

**Table 3:** Amino acid profile of practical white shrimp diets with different thermal energy input of sorghum and wheat.

Amino acid	Wheat_LE	Wheat_ME	Wheat_HE	Sorghum_LE	Sorghum_ME	Sorghum_HE
EAAs						
Arginine	2.21	2.21	2.18	2.17	2.16	2.22
Histidine	0.90	0.90	0.90	0.91	0.90	0.91
Isoleucine	1.67	1.65	1.66	1.72	1.68	1.68
Leucine	3.31	3.27	3.25	3.54	3.55	3.56
Lysine	2.00	1.99	1.99	1.99	1.97	2.00
Methionine	0.68	0.67	0.66	0.68	0.67	0.68
Phenylalanine	1.83	1.81	1.80	1.87	1.87	1.88
Threonine	1.33	1.31	1.32	1.35	1.35	1.36
Tryptophan	0.41	0.39	0.33	0.39	0.38	0.37
Valine	1.79	1.78	1.78	1.81	1.80	1.82
NEAAs						
Alanine	2.03	2.00	2.00	2.20	2.20	2.21
Aspartic acid	3.33	3.28	3.31	3.41	3.37	3.41
Cysteine	0.55	0.55	0.54	0.54	0.54	0.53
Glutamic acid	7.15	7.08	7.09	7.03	7.00	7.07
Glycine	1.73	1.71	1.70	1.70	1.69	1.69
Hydroxylysine	0.04	0.04	0.03	0.03	0.04	0.04
Hydroxyproline	0.18	0.16	0.16	0.17	0.16	0.17
Ornithine	0.03	0.03	0.03	0.03	0.03	0.03
Proline	2.06	2.02	2.03	2.06	2.04	2.05
Serine	1.54	1.51	1.51	1.57	1.58	1.60
Taurine	0.20	0.19	0.18	0.19	0.20	0.20
Tyrosine	1.27	1.30	1.20	1.29	1.31	1.38
Sum AA	36.24	35.85	35.65	36.65	36.49	36.86

*Note:* Analysis was performed according to the AOAC Official Method 982.30 E (a,b,c) chp. 45.3.05, 2006. Tryptophan was analyzed by alkaline hydrolysis—AOAC Official Method 988.15, chp. 45.4.04, 2006. Diets were made at Kansas State University. Results are expressed as g/100 g as is. Values represent means of two replicates. Analysis was performed by the University of Missouri.

Abbreviations: AA, amino acid; AOAC, Association of Official Agricultural Chemists; EAAs, essential amino acids; NEAAs, nonessential amino acids.

**Table 4:** Degree of gelatinization by the enzymatic method.

Diet	Degree of gelatinization (%)
Wheat_LE	94.56^bc^
Wheat_ME	98.75^a^
Wheat_HE	97.47^ab^
Sorghum_LE	83.00^d^
Sorghum_ME	85.07^d^
Sorghum_HE	90.49^c^
Two-way ANOVA	
Model	<0.0001
Process energy	0.0003
Grain	<0.0001
Process energy × grain	0.0075

*Note:* Means not sharing the same letter are significantly different by Tukey's HSD-test (parametric ANOVA) at the 5% level of significance, with letters representing the interaction between grain source and process energy. Analysis was done in accordance with AOAC 996.11 [22]. Values represent the means of three replicates. Analysis was performed by Midwest Laboratories, Inc., Omaha, NE, USA.

Abbreviations: ANOVA, analysis of variance; AOAC, Association of Official Agricultural Chemists.

**Table 5:** Water quality parameters of the experimental systems used for the growth and the digestibility trials.

Parameters	Growth trial	Digestibility trial
Dissolved oxygen (mg/L)	6.97 ± 0.33	7.09 ± 0.23
Temperature (°C)	28.08 ± 1.31	27.51 ± 0.71
Salinity (g/L)	7.27 ± 0.15	9.52 ± 0.67
Total ammonia nitrogen (mg/L)	0.04 ± 0.08	0.06 ± 0.04
Nitrite nitrogen (mg/L)	0.10 ± 0.05	0.10 ± 0.07
pH	8.10 ± 0.07	7.8 ± 0.00

**Table 6:** Response of white shrimp (mean initial weight 0.82 g ± 0.05) fed diets containing different process energy input of sorghum and wheat within a 6-week period.

Diets	Survival (%)	Final weight (g)	Weight gain (g)	Weight gain (%)	TGC	FCR	Gross energy (cal/g)	Energy retention (%)
Wheat_LE	84.44	9.02	8.20	999	0.23	1.47	4537	22.25
Wheat_ME	86.67	8.94	8.13	995	0.23	1.46	4407	21.62
Wheat_HE	81.11	9.10	8.27	1008	0.23	1.52	4403	21.49
*p*-Value	0.71	0.86	0.88	0.98	0.81	0.80	0.64	0.78
PSE	4.77	0.19	0.20	42.56	0.01	0.07	111.64	0.81
Sorghum_LE	84.44	8.79	7.96	963	0.23	1.49	4400	20.84
Sorghum_ME	83.33	8.84	8.02	976	0.23	1.51	4434	21.65
Sorghum_HE	80.00	9.26	8.44	1036	0.24	1.44	4434	21.76
*p*-Value	0.54	0.40	0.39	0.42	0.36	0.57	0.92	0.66
PSE	2.88	0.26	0.26	40.51	0.01	0.05	67.09	0.77
Two-way ANOVA								
Model	0.86	0.73	0.73	0.86	0.67	0.93	0.90	0.89
Ingredient	0.65	0.75	0.76	0.79	1.00	0.96	0.73	0.57
Process energy	0.48	0.39	0.39	0.57	0.35	1.00	0.83	0.99
Ingredient × process energy	0.91	0.70	0.68	0.73	0.60	0.52	0.59	0.52

*Note:* Values represent means of six replicates for experimental diets.

Abbreviations: ANOVA, analysis of variance; FCR, feed conversion ratio; TGC, thermal growth coefficient.

**Table 7:** Apparent digestibility coefficients of protein (APD), energy (AED) of the diet (D) offered to Pacific white shrimp, *Litopenaeus vannamei*.

Diet	AEDD	APDD
Wheat_LE	79.03 ± 1.23^Ab^	82.59 ± 1.07^Ac^
Wheat_ME	80.55 ± 0.38^Aab^	84.73 ± 0.65^Ab^
Wheat_HE	82.93 ± 1.34^Aa^	86.99 ± 0.52^Aa^
*p*-Value	0.01	0.001
PSE	0.62	0.45
Sorghum_LE	75.41 ± 0.52^Bab^	78.28 ± 0.56^B^
Sorghum_ME	74.29 ± 0.79^Bb^	77.79 ± 0.97^B^
Sorghum_HE	76.79 ± 1.00^Ba^	79.81 ± 1.75^B^
*p*-Value	0.02	0.18
PSE	0.46	0.69
Two-way ANOVA		
Model	<0.0001	<0.0001
Ingredient	<0.0001	<0.0001
Process energy	0.001	0.001
Ingredient × process energy	0.056	0.056

*Note:* The values from each diet are means and SD of triplicate tanks. Means within the same column not sharing any letter are significantly different by Tukey's HSD-test (parametric ANOVA) at the 5% level of significance, with uppercase and lowercase letters representing ingredient and process energy, respectively.

Abbreviations: AEDD, apparent energy digestibility coefficients; ANOVA, analysis of variance; APDD, apparent protein digestibility coefficients.

## Data Availability

The data that support the findings of this study are available from the corresponding author upon reasonable request.
